# Association of different iron deficiency cutoffs with adverse outcomes in chronic kidney disease

**DOI:** 10.1186/s12882-018-1021-3

**Published:** 2018-09-12

**Authors:** Michele F. Eisenga, Ilja M. Nolte, Peter van der Meer, Stephan J. L. Bakker, Carlo A. J. M. Gaillard

**Affiliations:** 1 Department of Internal Medicine, Division of Nephrology, University of Groningen, University Medical Center Groningen, Groningen, The Netherlands; 2 Department of Epidemiology, University of Groningen, University Medical Center Groningen, Groningen, The Netherlands; 3 Department of Cardiology, University of Groningen, University Medical Center Groningen, Groningen, The Netherlands; 4 Department of Internal Medicine and Dermatology, University of Utrecht, University Medical Center Utrecht, Utrecht, The Netherlands; 5 Department of Internal Medicine, Division of Nephrology, University Medical Center Groningen, P.O. Box 30.001, 9700 RB Groningen, The Netherlands

**Keywords:** Iron deficiency, CKD, Mortality, Cutoffs, Ferritin, TSAT

## Abstract

**Background:**

Iron deficiency is highly prevalent in chronic kidney disease (CKD) patients. In clinical practice, iron deficiency is defined based on a combination of two commonly used markers, ferritin and transferrin saturation (TSAT). However, no consensus has been reached which cutoffs of these parameters should be applied to define iron deficiency. Hence, we aimed to assess prospectively which cutoffs of ferritin and TSAT performed optimally for outcomes in CKD patients.

**Methods:**

We meticulously analyzed 975 CKD community dwelling patients of the Prevention of Renal and Vascular Endstage Disease prospective study based on an estimated glomerular filtration rate < 60 ml/min/1.73m^2^, albuminuria > 30 mg/24 h, or albumin-to-creatinine ratio ≥ 30 mg/g. Cox proportional hazard regression analyses using different sets and combinations of cutoffs of ferritin and TSAT were performed to assess prospective associations with all-cause mortality, cardiovascular mortality, and development of anemia.

**Results:**

Of the included 975 CKD patients (62 ± 12 years, 64% male with an estimated glomerular filtration rate of 77 ± 23 ml/min/1.73m^2^), 173 CKD patients died during a median follow-up of 8.0 (interquartile range 7.5–8.7) years of which 70 from a cardiovascular cause. Furthermore, 164 CKD patients developed anemia. The highest risk for all-cause mortality (hazard ratio, 2.83; 95% confidence interval, 1.53–5.24), cardiovascular mortality (4.15; 1.78–9.66), and developing anemia (3.07; 1.69–5.57) was uniformly observed for a TSAT< 10%, independent of serum ferritin level.

**Conclusion:**

In this study, we have shown that of the traditionally used markers of iron status, reduced TSAT, especially TSAT< 10%, is most strongly associated with the risk of adverse outcomes in CKD patients irrespective of serum ferritin level, suggesting that clinicians should focus more on TSAT rather than ferritin in this patient setting. Specific attention to iron levels below this cutoff seems warranted in CKD patients.

**Electronic supplementary material:**

The online version of this article (10.1186/s12882-018-1021-3) contains supplementary material, which is available to authorized users.

## Background

Iron deficiency (ID) is the most common nutritional deficiency worldwide, affecting up to 25% of the population [1–3]. A variety of causes are responsible for the depletion of iron stores, ranging from deficient dietary iron intake to increased blood loss (e.g. gastro-intestinal cancers, peptic ulcera) [4, 5]. In addition, in chronic disease populations, due to the pro-inflammatory state that chronic diseases constitute, upregulation of serum hepcidin blocks iron absorption from the gut and iron release from the reticulo-endothelial system leading to reduced iron availability despite adequate stores [6]. Indeed, in these populations, such as chronic heart failure (CHF) and chronic kidney disease (CKD), it has been shown that ID is highly prevalent and associated with an increased risk of morbidity and mortality, independent of potential confounders, including anemia [7–9].

The definition of ID is still a matter of debate [10, 11]. ID is generally divided into absolute ID (low iron stores) and functional ID (insufficient iron supply to the bone marrow despite sufficient iron stores). Due to the existence of both absolute ID and functional ID and the absence of an unequivocal gold standard, it remains challenging to correctly identify ID [12]. Clinicians and epidemiologists alike predominantly rely on two frequently used markers, namely ferritin (for iron load) and transferrin saturation (TSAT, for iron transport availability) [13–15]. However, to date, no consensus has been reached which cutoffs of these parameters should be utilized to define absolute and functional ID per population. Except perhaps in the cardiology field where absolute ID is defined as a ferritin level < 100 μg/L, and functional ID as a TSAT< 20% accompanied by ferritin levels between 100 and 299 μg/L [7, 16]. Currently in nephrology, the Kidney Disease Improving Global Outcomes (KDIGO) committee recommends a trial of 1 to 3 months of oral iron therapy in non-dialysis CKD patients when TSAT levels are below 30% and ferritin below 500 μg/L. However, it is not known which cutoffs of ferritin and/or TSAT perform best with respect to predicting anemia, response to iron treatment or outcome [17].

Correctly defining which cutoffs of ferritin and TSAT associate with outcome would identify which patients are most at risk to develop these outcomes and thus in which patients correction of ID could potentially have the greatest benefit. Therefore, the present study was performed to define which cutoffs of serum ferritin and TSAT perform optimally for the risk of all-cause mortality, cardiovascular mortality, and risk of developing anemia in CKD patients.

## Methods

### Study population

**Fig. 1:**
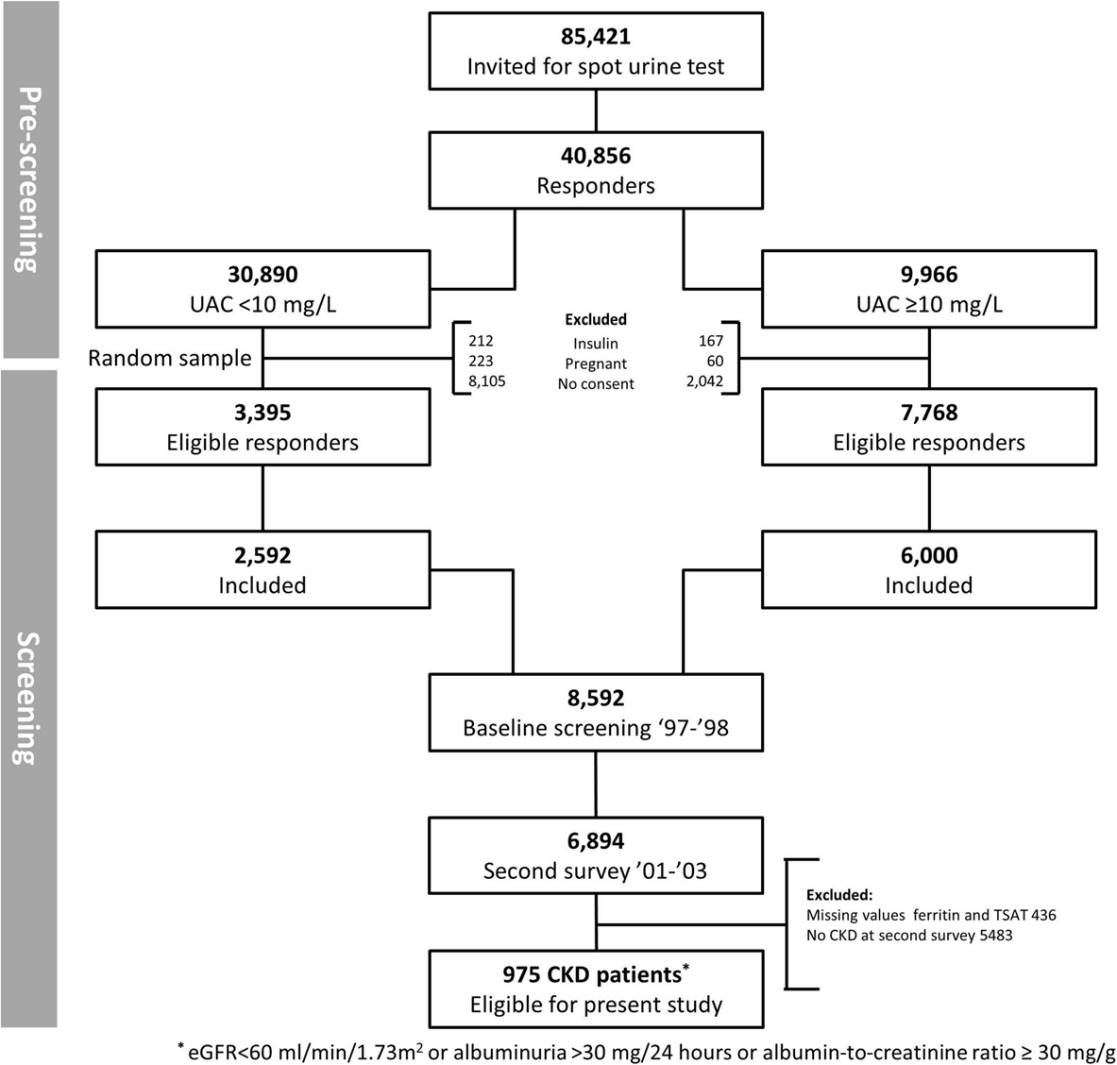
Flowchart of the participants in the study

Data was used from the Prevention of Renal and Vascular End-Stage Disease (PREVEND) study, of which details have been published elsewhere [18]. In brief, from 1997 to 1998, all inhabitants of the city of Groningen, The Netherlands, ranging between 28 and 75 years of age, received a questionnaire to complete regarding demographics, disease history, smoking status, medication use, and a vial to collect a first morning urine sample (*n* = 85,421). Eventually, 40,856 subjects responded on the request (47.8%). We excluded subjects with type 1 diabetes mellitus (defined as the use of insulin) and pregnant women. After completion of the screening protocol, subjects with an urinary albumin excretion (UAE) ≥10 mg/L (*n* = 6000) and a randomly selected control group with an UAE < 10 mg/L (*n* = 2592) formed the baseline PREVEND cohort (*n* = 8592). For current analyses, we used data from the second survey, which occurred between 2001 and 2003 (*n* = 6894), due to the availability of iron status parameters at this visit. Participants visited the outpatient clinic twice and were requested to collect two consecutive 24-h urine specimens. We excluded 436 subjects due to missing values for serum ferritin or serum transferrin, resulting in the inclusion of 6458 subjects. For current analyses, we selected all patients with CKD, based on an eGFR< 60 ml/min/1.73m^2^ or albuminuria > 30 mg/24 h or albumin-to-creatinine ratio ≥ 30 mg/g (*n* = 975, flowchart depicted in Fig. 1). As sensitivity analyses, we restricted the CKD patients group to patients with solitarily an eGFR< 60 ml/min/1.73m^2^ (*n* = 274). The PREVEND study protocol was approved by the institutional medical review board and was carried out in accordance with the Declaration of Helsinki. Written informed consent was obtained from all participants.

### Measurements

Fasting blood samples were drawn in the morning from all participants from 2001 to 2003. All hematologic measurements were measured in fresh venous blood. Aliquots of these samples were stored immediately at − 80 °C until further analysis. Measurements were performed at the central laboratory of the University Medical Center Groningen. Serum creatinine was measured using an enzymatic, IDMS-traceable method on a Roche Modular analyzer (Roche Diagnostics, Mannheim, Germany). For estimating glomerular filtration rate (eGFR), the Chronic Kidney Disease Epidemiology Collaboration (CKD-EPI) was applied [19]. In the PREVEND study, serum iron (μmol/L), ferritin (μg/L), and transferrin (g/L) were measured using a colorimetric assay, immunoassay, and immunoturbidimetric assay, respectively (Roche Diagnostics).

### Main outcomes

We assessed the association of ferritin and TSAT with all-cause mortality, cardiovascular mortality and risk of developing anemia. In the PREVEND cohort, data on mortality were received through the municipal register. Cause of death was gathered by combining the number reported on the death certificate with the primary cause of death as classified by the Dutch Central Bureau of Statistics. Anemia was defined as a hemoglobin level lower than 7.5 mmol/L (12 g/dL) for women, and a hemoglobin level lower than 8.1 mmol/L (13 g/dL) for men [20]. Follow-up was performed until the 1st of January 2011 in the PREVEND study.

### Statistical analyses

Baseline variables are described by means with SD when variables are continuous and normally distributed, by medians with interquartile range when the distribution is skewed, or as numbers and corresponding percentages for categorical data. Differences between groups were assessed with a Student’s t-test for normally distributed variables, a Mann-Whitney U-test for skewed variables, and a Chi-square test for categorical variables. We evaluated the presence of high ferritin levels (suggestive of iron trapping) despite a functional iron deficiency by calculating the percentage of CKD patients with ferritin levels higher than 500 μg/L in combination with a TSAT< 20%. Cox regression analyses were performed to investigate the hazard rates of the individual cutoffs of ferritin and TSAT and all combinations of serum ferritin (i.e. < 20, < 50, < 100, < 200, < 300, and < 500 μg/L) and TSAT (i.e. < 10, < 15, < 20, < 25, and < 30%) for the respective outcomes while adjusting for age and sex. Separately, we also assessed conditional definitions as defined in the Ferinject Assessment in Patients with Iron Deficiency and Chronic Heart Failure (FAIR-HF) study, i.e. ferritin< 100 μg/L or TSAT< 20% with ferritin < 300 μg/L, and in the Ferinject in patients with Iron deficiency anemia and Non-Dialysis-dependent Chronic Kidney Disease (FIND-CKD) study, i.e. ferritin< 100 μg/L or TSAT< 20% with ferritin < 200 μg/L [7, 21]. Based on these two conditional definitions, we extended the possibilities of conditional definitions with a TSAT lower than 10% and lower than 15% in combination with a ferritin level < 200 μg/L or < 300 μg/L. To really allow identification of the group with the highest risk based on cutoffs, in each analysis the reference group is defined as the group above the studied cutoff, as a result the reference group in the Cox regression analyses is variable, e.g. when the combined definition of TSAT< 20% with ferritin < 300 μg/L was analyzed, the reference group was TSAT> 20% with ferritin> 300 μg/L. Cutoffs were chosen as rounded numbers rather than subdividing TSAT and ferritin levels in quartiles or quintiles to allow comparison with cutoffs generally chosen in clinical practice and research studies. As sensitivity analyses, we performed subanalyses in 274 CKD patients with solitarily an eGFR lower than 60 ml/min/1.73m^2^, and in 717 CKD patients with data available on hs-CRP, as inflammation marker, and on serum albumin, as proxy for malnutrition. Finally, we made splines of ferritin and TSAT with all-cause mortality using restricted cubic splines based on Cox regression proportional hazard analyses. Data were analyzed using IBM SPSS software, version 23.0 (SPSS Inc., Chicago, IL), and R version 3.2.3 (Vienna, Austria).

## Results

### Patient characteristics

We included 975 CKD patients (62 ± 12 years, 64% male) with a mean eGFR of 77 ± 23 ml/min/1.73m^2^. Further demographics and clinical characteristics of the CKD patients, dichotomized according to survival status at the end of the follow-up are shown in Table 1. High ferritin levels of > 500 μg/L (representing possibly iron overload) in combination with functional iron deficiency (TSAT< 20%) was present in 6 (0.6%) of the 975 CKD patients.

**Table 1:** Baseline characteristics of 975 chronic kidney disease patients

Characteristic	TSAT (%)				Ferritin (μg/L)			
	< 10 [*n* = 41]	10- < 20 [*n* = 278]	20- < 30 [*n* = 414]	≥30 [*n* = 242]	< 50 [*n* = 169]	50- < 100 [*n* = 219]	100- < 300 [*n* = 453]	≥300 [*n* = 134]
Demographics								
Age, yr	60 ± 12	63 ± 12	62 ± 11	63 ± 11	59 ± 13	63 ± 11	63 ± 11	64 ± 11
Male sex – no. (%)	17 (42)	151 (54)	277 (67)	180 (74)	59 (35)	136 (62)	317 (70)	113 (84)
Race (n, %)	37 (90)	263 (95)	397 (96)	235 (97)	159 (94)	213 (97)	437 (97)	123 (92)
BMI, kg/m^2^	27.6 ± 4.8	29.4 ± 5.7	28.3 ± 4.3	27.4 ± 4.1	26.9 ± 5.6	27.9 ± 4.7	28.7 ± 4.6	29.5 ± 4.0
Systolic blood pressure, mmHg	135 ± 24	140 ± 22	139 ± 22	139 ± 22	133 ± 21	138 ± 21	140 ± 22	145 ± 21
Diabetes Mellitus, n (%)	6 (15)	67 (24)	69 (17)	36 (15)	23 (14)	27 (12)	99 (22)	29 (22)
Current smoking, n (%)	6 (15)	76 (28)	121 (29)	72 (30)	45 (27)	69 (32)	127 (28)	34 (25)
Alcohol use, n(%)	22 (54)	176 (63)	298 (72)	181 (75)	99 (59)	147 (67)	329 (73)	102 (76)
Baseline renal function								
Creatinine (μmol/L)	85 (73–113)	90 (79–104)	92 (81–107)	95 (80–106)	85 (74–99)	92 (79–107)	93 (83–108)	93 (84–109)
eGFR (ml/min/1.73m^2^)	78 ± 26	76 ± 23	78 ± 22	78 ± 22	81 ± 26	76 ± 23	76 ± 22	77 ± 23
CKD stage								
Stage I (n, %)	12 (29)	84 (30)	136 (33)	71 (29)	56 (33)	70 (32)	130 (29)	47 (35)
Stage II (n, %)	13 (32)	97 (35)	140 (34)	99 (41)	59 (35)	72 (33)	174 (38)	44 (33)
Stage III (n, %)	10 (24)	76 (27)	114 (28)	60 (25)	37 (22)	62 (28)	126 (28)	35 (26)
Stage I*V*/V (n, %)	1 (2)	7 (3)	3 (1)	3 (1)	2 (1)	2 (1)	7 (2)	3 (2)
Urinary albumin excretion (mg/24 h)	51 (36–139)	53 (33–140)	54 (34–111)	56 (36–112)	48 (33–104)	54 (33–107)	57 (34–128)	63 (38–121)
Laboratory parameters								
Hemoglobin (g/dL)	12.0 ± 2.1	13.6 ± 1.3	14.2 ± 1.2	14.4 ± 1.0	13.0 ± 0.6	13.9 ± 1.1	14.2 ± 1.2	14.5 ± 1.4
MCV (fL)	82 ± 8	89 ± 5	91 ± 4	92 ± 4	88 ± 7	91 ± 4	91 ± 4	92 ± 6
Ferritin (μg/L)	16 (8–28)	102 (56–175)	132 (72–235)	168 (101–276)	29 (17–40)	74 (62–87)	167 (130–214)	417 (343–553)
TSAT (%)	7 ± 2	16 ± 2	24 ± 3	37 ± 7	18 ± 2	24 ± 2	26 ± 9	29 ± 11
Serum iron (μmol/L)	6 ± 2	11 ± 2	16 ± 3	22 ± 4	13 ± 5	15 ± 5	16 ± 5	17 ± 6
hs-CRP (mg/dL)^a^	2.7 (1.1–7.9)	4.1 (1.7–7.0)	2.1 (1.1–4.1)	2.0 (1.1–3.9)	2.0 (0.9–5.1)	2.2 (1.2–5.1)	2.6 (1.3–4.7)	2.4 (1.5–4.9)
Albumin (g/L)^a^	42 ± 2	43 ± 3	43 ± 3	44 ± 3	43 ± 3	43 ± 3	44 ± 3	44 ± 3
Total cholesterol (mmol/L)	5.1 ± 1.2	5.5 ± 1.2	5.5 ± 1.0	5.5 ± 1.0	5.2 ± 1.2	5.5 ± 1.1	5.5 ± 1.1	5.7 ± 1.2
Glucose (mmol/L)	5.8 ± 2.1	5.9 ± 1.9	5.5 ± 1.5	5.5 ± 1.6	5.4 ± 1.8	5.3 ± 1.3	5.8 ± 1.7	5.9 ± 1.8

Normally distributed variables are shown as mean with standard deviation, whereas skewed variables are shown as medians with interquartile range. ^a^Albumin and hs-CRP data were available in 717 patients

**Fig. 2:**
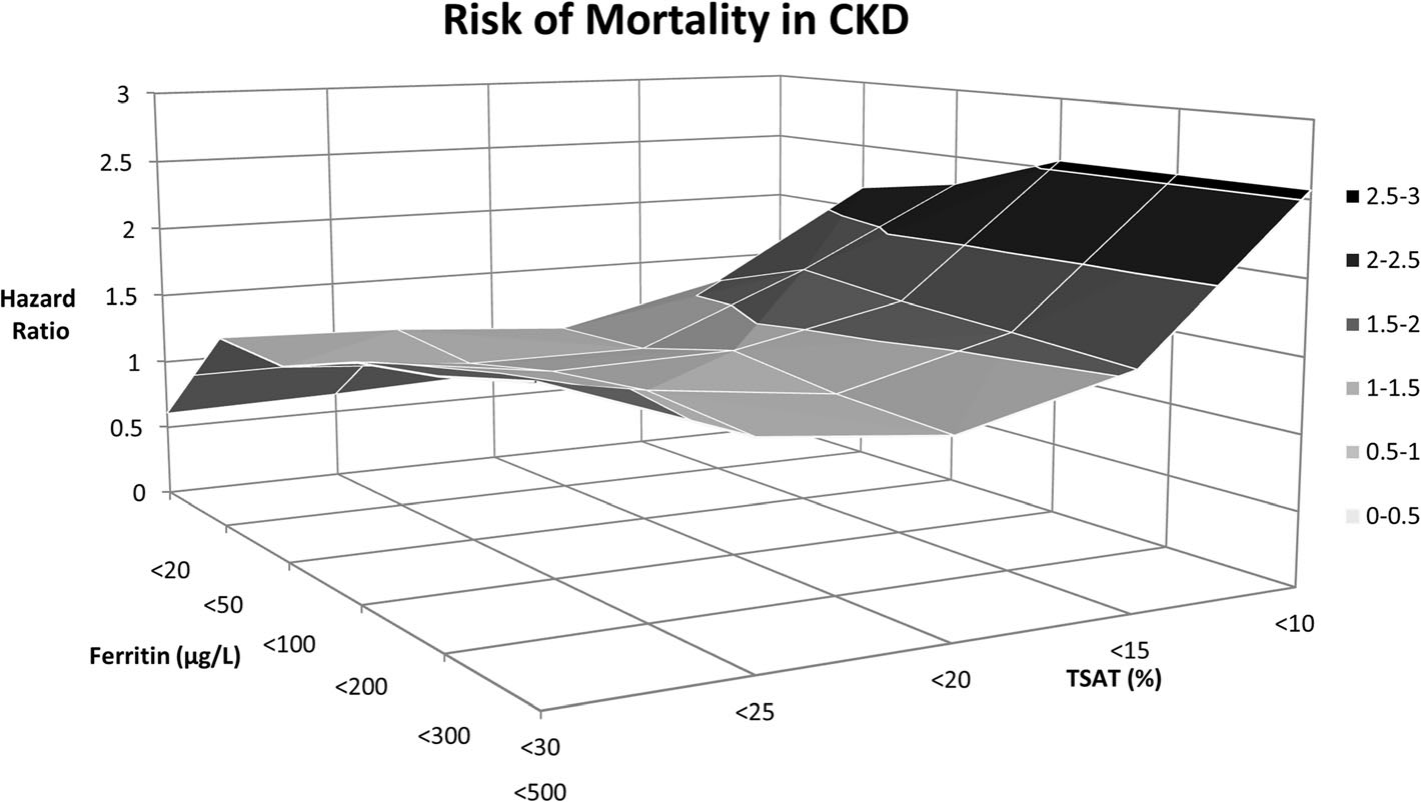
Hazard ratio of mortality risk by different cutoff values of ferritin and TSAT in chronic kidney disease patients

**Fig. 3:**
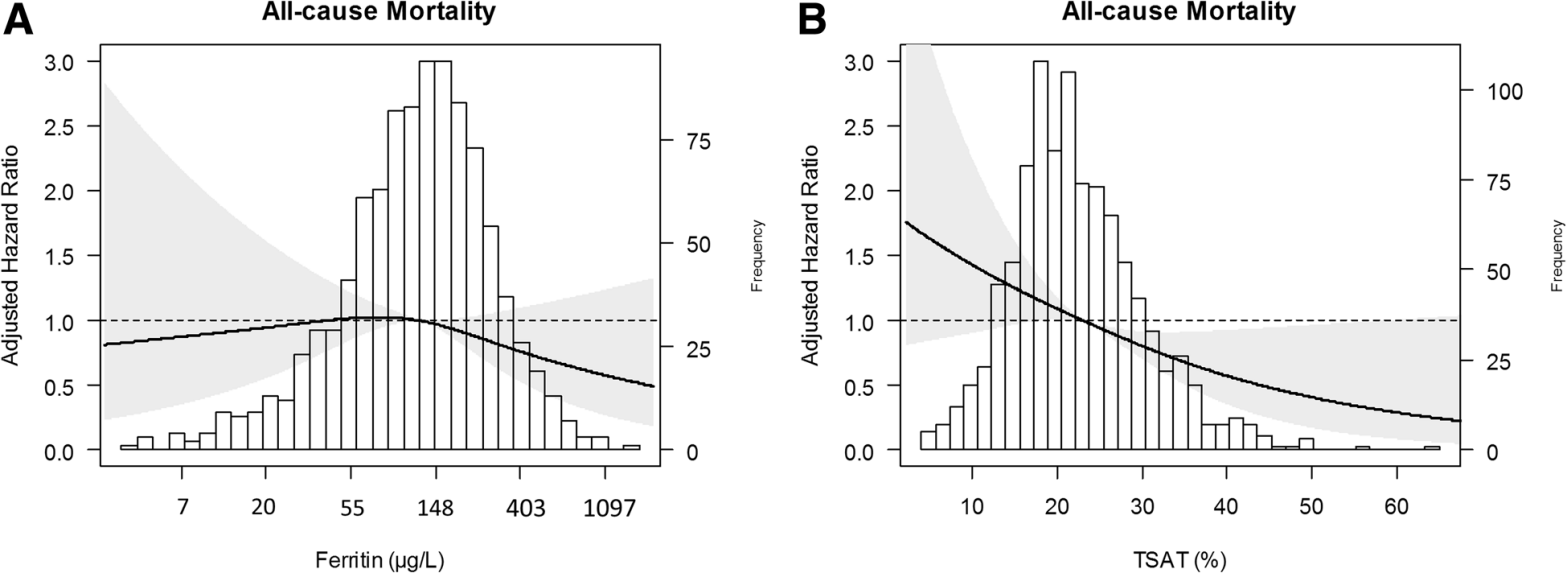
Associations of TSAT and ferritin as continuous variables on all-cause mortality in chronic kidney disease patients. Data were fit by a Cox proportional hazard regression model based on restricted cubic splines. Knots were placed on at 10th, 50th, and 90th percentile of ferritin and TSAT, respectively. Panel **a** shows the association between ferritin, adjusted for age and sex, and all-cause mortality. Panel **b** shows the association between TSAT, adjusted for age and sex, and all-cause mortality. The black line represents the hazard ratio. The grey area the 95% confidence interval

First, we assessed the impact of using different cutoffs for ferritin or TSAT as individual markers on the association with all-cause mortality. During a median follow-up of 8.0 (interquartile range 7.5–8.7) years, 173 CKD patients died. The highest age- and sex-adjusted risk of mortality (HR, 2.83; 95%CI 1.53–5.24) was observed for TSAT< 10%. Ferritin, as individual continuous marker, was not associated with increased risk of mortality. When assessing the impact of using a different combination of ferritin and TSAT on mortality, the highest risk of mortality (HR, 2.56; 95%CI 1.35–4.87) was observed for TSAT< 10% in combination with ferritin cutoffs of either < 200, 300, or 500 μg/L (Fig. 2). Full analyses are shown in Additional file 1. Using a conditional definition did not improve the observed maximal HR. In restricted cubic splines, these findings are further illustrated as the same pattern was observed for continuous variables of ferritin (Fig. 3a) and TSAT (Fig. 3b).

Second, we assessed the impact of using different cutoffs for ferritin or TSAT as individual markers on the association with cardiovascular mortality. Of the 173 deceased CKD patients, 70 were due to cardiovascular cause. The highest risk of cardiovascular mortality (HR, 4.15; 95%CI 1.78–9.66) was observed for TSAT< 10%. Ferritin, as individual continuous marker, was not associated with increased risk of cardiovascular mortality. When assessing the impact of using a different combination of ferritin and TSAT on mortality, the highest risk of cardiovascular mortality (HR, 4.17; 95%CI 1.79–9.70) was identified for TSAT< 10% in combination with a ferritin cutoff of < 100, 200, 300, or 500 μg/L (Additional file 2).

Third, we examined the impact of using different cutoffs for ferritin or TSAT as individual markers for the risk of developing anemia. During median follow-up of 6.2 (2.4–7.5) years, 164 CKD patients developed anemia. The highest risk of developing anemia (HR, 3.07; 95%CI 1.69–5.57) was observed for TSAT < 10%. Ferritin as individual continuous marker had the highest risk of anemia with a cutoff of ferritin< 20 μg/L (HR, 2.95; 95%CI 1.75–4.98). In the various combinations of ferritin with TSAT, the highest risk of developing anemia (HR, 3.07; 95%CI 1.69–5.57) was identified for TSAT< 10% in combination with a ferritin cutoff of < 100, 200, 300, or 500 μg/L. All the other combinations were also positively associated with development of anemia albeit less significant (Additional file 3).

As sensitivity analyses, we restricted the group of 975 CKD patients to 274 CKD patients with an eGFR lower than 60 ml/min/1.73m^2^. We repeated the analyses of the different cutoffs of ferritin and/or TSAT with all-cause mortality, cardiovascular mortality, and risk of anemia. In these analyses, we identified the same pattern that the highest risk was particularly observed for a TSAT< 10% (Additional file 4).

Furthermore, we performed sensitivity analyses in 717 CKD patients of the included 975 CKD patients with data available on hs-CRP and serum albumin. We repeated all the previous analyses on the impact of using different cutoffs for ferritin and/or TSAT on the association with all-cause mortality, cardiovascular mortality, and risk of anemia. In these analyses, we identified again the same pattern, i.e. that the highest risk was particularly observed for a TSAT< 10% (Additional file 5).

## Discussion

In this study, consisting of a large cohort of CKD patients, we show the impact of using different cutoffs for ferritin and/or TSAT on the association with all-cause mortality, cardiovascular mortality, and the subsequent development of anemia. Remarkably, in CKD patients the highest risk to develop adverse outcomes was uniformly observed at a low TSAT level, i.e. lower than 10%, largely independent of the level of ferritin. The current results are of importance for defining ID in CKD patients and may aid clinicians to focus on these specific cutoffs for TSAT in order to improve outcome.

To date, there is no consensus in the field of CKD which cutoffs of ferritin and TSAT should be retained to define ID. Multiple important studies in CKD patients have utilized different definitions for ID. For example, the FIND-CKD study used serum ferritin < 100 μg/L or TSAT < 20% in combination with serum ferritin of < 200 μg/L to identify patients as iron deficient, whereas Qunibi and colleagues defined ID as TSAT ≤25% with ferritin ≤300 μg/L and Fishbane and colleagues utilized TSAT ≤25% in combination with ferritin ≤200 μg/L to determine ID [21–23]. Currently, the plethora of ID definitions impedes comparability among iron studies. As a result, translation to clinical practice is difficult. Therefore, it is important to identify optimal cutoffs for CKD patients. Accordingly, in the present study, we assessed prospectively which cutoffs for ferritin and TSAT performed optimally for the association with adverse outcomes, implicating that, at least in terms of survival and development of anemia, these selected cutoffs are clinically most relevant.

Previously, few studies have evaluated the accuracy of serum ferritin and TSAT cutoffs to define ID in CKD patients in terms of sensitivity and specificity. Fishbane et al. determined in hemodialysis (HD) patients which levels of serum ferritin and TSAT were most predictive for ID. The authors concluded that in erythropoietin-responsive patients ferritin level of lower than 100 μg/L or TSAT< 18% are indicative of inadequate iron status, whereas in erythropoietin-resistant patients a serum ferritin < 300 μg/L or a TSAT < 27% should be utilized [24]. Also in HD patients, Kalantar-Zadeh et al. identified high specificity for a cutoff of serum ferritin < 200 ng/mL and high sensitivity for a TSAT< 20% [25]. As far as we know, we are the first in CKD patients to assess the performance of different cutoffs for ferritin and/or TSAT in terms of prospective associations with adverse outcomes.

Our results identified TSAT lower than 10% to be the optimal cutoff associated with increased risk of detrimental outcomes in the CKD population. In our population of early stage CKD patients, i.e. CKD stadia one to three, the importance of adequate iron status is evident, in view of the increased hazard ratios for development of adverse outcomes. When carefully assessing the hazard ratios for all-cause mortality, cardiovascular mortality, and risk of anemia, it is clear that the highest risk is observed for TSAT< 10%, however, also for TSAT< 15% a significant increased risk in adverse outcomes is observed. It should be noted that the cardiovascular mortality risk associated with ID is markedly higher (nearly double) than the risk for all-cause mortality. For a cutoff value of TSAT < 20 and < 30% the observed hazard ratios for all-cause mortality and cardiovascular mortality are less impressive, whereas the risk for anemia decreases steadily with increasing cutoff levels. Conditional definitions as those used previously in the FAIR-HF and FIND-CKD did not improve the association with increased risk. This suggests that in CKD patients the main focus should be on low TSAT, especially TSAT lower than 10%. Based on these results, it may be speculated that failure to correct these low TSAT levels might jeopardize the survival of CKD patients.

Currently, ferritin and TSAT are the most commonly used markers in clinical setting to evaluate iron stores and iron availability. However, there are important drawbacks on the use of ferritin and TSAT as iron status parameters. Serum ferritin is an acute-phase reactant and therefore in chronic disease populations serum ferritin levels will be elevated [26, 27]. TSAT also has acute-phase reactivity as transferrin is elevated in the setting of acute inflammation which will lower TSAT when circulating iron remains constant [28]. However, other markers, such as soluble transferrin receptor, percentage hypochromic red blood cells, and reticulocyte hemoglobin content, are not readily available in clinical practice, less well studied, or not used for other reasons.

Our study has strengths and limitations. Strengths are that it comprises a large cohort of CKD patients with availability of data on iron status and that it is the first study to assess all combinations of cutoffs with respect to “hard” clinical endpoints. Limitations of the current study include its observational design, that it comprises a single center study and that measurement of iron parameters were performed at a single time point, which precludes our ability to discern the impact of changes in iron parameters over time on clinical outcomes. Furthermore, the current study is only valid for early CKD, and precludes us to discern whether similar results apply for more advanced CKD stages. Another limitation might be that we did not adjust for several potential confounders in the different associations between ID and outcomes, however, the primary aim of this study is to study the prospective associations of ID with adverse outcomes using several cutoffs for ferritin and TSAT, not to investigate the mechanisms involved.

## Conclusion

In this study, we show that in CKD patients the highest risk for adverse outcomes with ID is observed when for the definition of ID a TSAT cutoff level lower than 10% is used. The use of the TSAT cutoff is largely independent of the level of serum ferritin. This suggests that emphasis should be placed on a low TSAT rather than ferritin levels in early stage CKD patients. Further research is needed to validate our results in terms of the effect of iron treatment on outcomes.

## Additional files

Additional file 1: **Table S1.** Different cutoff values of ferritin and TSAT, adjusted for age and sex, with respect to risk of all-cause mortality in 975 CKD patients (based on eGFR< 60 ml/min/1.73m^2^ or albuminuria > 30 mg/24 h or albumin-to-creatinine ratio ≥ 30 mg/g). (DOCX 16 kb)

Additional file 2: **Table S2.** Association of different cutoff values of ferritin and TSAT, adjusted for age and sex, with respect to risk of cardiovascular mortality in CKD patients (based on eGFR< 60 ml/min/1.73m^2^ or albuminuria > 30 mg/24 h or albumin-to-creatinine ratio ≥ 30 mg/g). (PDF 229 kb)

Additional file 3: **Table S3.** Association of different cutoff values of ferritin and TSAT, adjusted for age and sex, with respect to risk of anemia in CKD patients (based on eGFR< 60 ml/min/1.73m^2^ or albuminuria > 30 mg/24 h or albumin-to-creatinine ratio ≥ 30 mg/g). (PDF 226 kb)

Additional file 4: **Table S4**, **Table S5**, and **Table S6.** Showing the association of different cutoff values of ferritin and TSAT, adjusted for age and sex, in CKD patients with eGFR< 60 ml/min/1.73m^2^ with respect to risk of all-cause mortality, cardiovascular mortality, and anemia, respectively. (PDF 195 kb)

Additional file 5: **Table S7**, **Table S8**, and **Table S9.** Showing the association of different cutoff values of ferritin and TSAT, adjusted for age, sex, hs-CRP, and albumin, in 717 CKD patients (based on eGFR< 60 ml/min/1.73m^2^ or albuminuria > 30 mg/24 h or albumin-to-creatinine ratio ≥ 30 mg/g) with respect to risk of all-cause mortality, cardiovascular mortality, and anemia, respectively. (PDF 255 kb)

## Abbreviations

CI: Confidence interval
CKD: Chronic kidney disease
CKD-EPI: Chronic Kidney Disease Epidemiology Collaboration
eGFR: Estimated glomerular filtration rate
HD: Hemodialysis
HR: Hazard ratio
ID: Iron deficiency
SD: Standard deviation
TSAT: Transferrin saturation
UAE: Urinary albumin excretion
