# Supplementary material for: Association of different iron deficiency cutoffs with adverse outcomes in chronic kidney disease
Source: BMC Nephrol. 2018 Sep 12;19:225. doi: 10.1186/s12882-018-1021-3 (PMC6134584; doi:10.1186/s12882-018-1021-3)
Supplement: Supplementary file 1 — Table S1. Different cutoff values of ferritin and TSAT, adjusted for age and sex, with respect to risk of all-cause mortality in 975 CKD patients (based on eGFR< 60 ml/min/1.73m2 or albuminuria > 30 mg/24 h or albumin-to-creatinine ratio ≥ 30 mg/g). (DOCX 16 kb) [file 12882_2018_1021_MOESM1_ESM.docx]

**Table S1.** Different cutoff values of ferritin and TSAT, adjusted for age and sex, with respect to risk of all-cause mortality in 975 CKD patients (based on eGFR<60 ml/min/1.73m^2^ or albuminuria >30 mg/24 hours or albumin-to-creatinine ratio ≥ 30 mg/g)

| **TSAT (%)** | **HR (95%CI)** |
| --- | --- |
| <10 | **2.83 (1.53-5.24)** |
| <15 | **1.57 (1.05-2.34)** |
| <20 | 1.25 (0.91-1.71) |
| <25 | 1.35 (0.99-1.85) |
| <30 | **1.87 (1.26-2.77)** |

| **Ferritin (µg/L)** | **HR (95%CI)** |
| --- | --- |
| <20 | 0.61 (0.19-1.92) |
| <50 | 1.24 (0.80-1.92) |
| <100 | 1.10 (0.81-1.50) |
| <200 | 1.24 (0.88-1.73) |
| <300 | 1.12 (0.74-1.71) |
| <500 | 1.66 (0.68-4.04) |

| **AND FERRITIN**  **TSAT** | <20 | <50 | <100 | <200 | <300 | <500 |
| --- | --- | --- | --- | --- | --- | --- |
| <10 | 0.81 (0.20-3.27) | **2.16 (1.01-4.62)** | **2.29 (1.17-4.50)** | **2.56 (1.35-4.87)** | **2.56 (1.35-4.87)** | **2.56 (1.35-4.87)** |
| <15 | 0.57 (0.14-2.32) | 1.49 (0.81-2.75) | 1.72 (1.07-2.77) | 1.64 (1.09-2.48) | 1.59 (1.06-2.40) | 1.53 (1.02-2.29) |
| <20 | 0.69 (0.22-2.18) | 1.22 (0.72-2.08) | 1.24 (0.84-1.83) | **1.40 (1.01-1.93)** | 1.30 (0.95-1.80) | 1.25 (0.91-1.72) |
| <25 | 0.64 (0.20-2.02) | 1.31 (0.82-2.11) | 1.24 (0.89-1.72) | **1.37 (1.02-1.85)** | **1.44 (1.06-1.95)** | **1.37 (1.01-1.88)** |
| <30 | 0.61 (0.20-1.93) | 1.35 (0.87-2.09) | 1.33 (0.98-1.82) | **1.54 (1.13-2.10)** | **1.64 (1.17-2.29)** | **1.80 (1.23-2.64)** |

**Conditional definitions:**

| Ferritin <100 µg/L or TSAT <10% with ferritin 100-199 µg/L | 1.18 (0.97-1.44) |
| --- | --- |
| Ferritin <100 µg/L or TSAT <10% with ferritin 100-299 µg/L | 1.18 (0.97-1.44) |
| Ferritin <100 µg/L or TSAT <15% with ferritin 100-199 µg/L | 1.19 (0.98-1.45) |
| Ferritin <100 µg/L or TSAT <15% with ferritin 100-299 µg/L | 1.19 (-HF))0.98-1.45) |
| Ferritin <100 µg/L or TSAT <20% with ferritin 100-199 µg/L (FIND-CKD) | 1.28 (0.95-1.72) |
| Ferritin <100 µg/L or TSAT <20% with ferritin 100-299 µg/L (FAIR-HF) | 1.19 (0.88-1.61) |
