# Supplementary material for: Association of different iron deficiency cutoffs with adverse outcomes in chronic kidney disease
Source: BMC Nephrol. 2018 Sep 12;19:225. doi: 10.1186/s12882-018-1021-3 (PMC6134584; doi:10.1186/s12882-018-1021-3)
Supplement: Supplementary file 2 — Table S2. Association of different cutoff values of ferritin and TSAT, adjusted for age and sex, with respect to risk of cardiovascular mortality in CKD patients (based on eGFR< 60 ml/min/1.73m2 or albuminuria > 30 mg/24 h or albumin-to-creatinine ratio ≥ 30 mg/g). (PDF 229 kb) [file 12882_2018_1021_MOESM2_ESM.pdf]

Supplemental Table 2. Different cutoff values of ferritin and TSAT, adjusted for age and sex, with respect to risk of cardiovascular mortality in CKD patients (based on eGFR<60 ml/min/1.73m^2 or albuminuria >30 mg/24 hours or albumin-to-creatinine ratio ≥ 30 mg/g)

**TSAT (%)**	**HR (95%CI)**
<10	**4.15 (1.78-9.66)**
<15	1.79 (0.98-3.27)
<20	1.28 (0.78-2.10)
<25	1.10 (0.69-1.78)
<30	1.88 (1.01-3.51)

**Ferritin (μg/L)**	**HR (95%CI)**
<20	0.52 (0.07-3.77)
<50	1.09 (0.54-2.21)
<100	1.12 (0.69-1.81)
<200	1.55 (0.88-2.72)
<300	1.53 (0.73-3.20)
<500	3.51 (0.49-25.28)

**TSAT**	**AND FERRITIN** <20	<50	<100	<200	<300	<500
<10	1.09 (0.15-7.99)	**3.31 (1.20-9.20)**	**4.17 (1.79-9.70)**	**4.16 (1.79-9.69)**	**4.16 (1.79-9.69)**	**4.16 (1.79-9.69)**
<15	0.76 (0.10-5.84)	**2.08 (0.90-4.81)**	**2.07 (1.03-4.18)**	**1.85 (0.99-3.45)**	1.81 (0.97-3.37)	1.81 (0.99-3.31)
<20	0.60 (0.08-4.33)	1.39 (0.63-3.05)	1.14 (0.61-2.13)	1.57 (0.95-2.58)	1.40 (0.85-2.30)	1.32 (0.81-2.16)
<25	0.55 (0.08-4.01)	1.27 (0.59-2.61)	1.07 (0.63-1.81)	1.17 (0.73-1.88)	1.26 (0.79-2.03)	1.15 (0.71-1.85)
<30	0.52 (0.07-3.87)	1.18 (0.58-2.40)	1.36 (0.84-2.22)	**1.74 (1.06-2.86)**	**1.75 (1.02-3.00)**	**2.05 (1.10-3.82)**

Conditional definitions:

Ferritin <100 μg/L or TSAT <10% with ferritin 100-199 μg/L	1.09 (0.75-1.58)
Ferritin <100 μg/L or TSAT <10% with ferritin 100-299 μg/L	1.09 (0.75-1.58)
Ferritin <100 μg/L or TSAT <15% with ferritin 100-199 μg/L	1.12 (0.77-1.63)
Ferritin <100 μg/L or TSAT <15% with ferritin 100-299 μg/L	1.11 (0.77-1.61)
Ferritin <100 μg/L or TSAT <20% with ferritin 100-199 μg/L (FIND-CKD)	1.33 (0.92-1.92)
Ferritin <100 μg/L or TSAT <20% with ferritin 100-299 μg/L (FAIR-HF)	1.38 (0.86-2.22)
