# Supplementary material for: Association of different iron deficiency cutoffs with adverse outcomes in chronic kidney disease
Source: BMC Nephrol. 2018 Sep 12;19:225. doi: 10.1186/s12882-018-1021-3 (PMC6134584; doi:10.1186/s12882-018-1021-3)
Supplement: Supplementary file 3 — Table S3. Association of different cutoff values of ferritin and TSAT, adjusted for age and sex, with respect to risk of anemia in CKD patients (based on eGFR< 60 ml/min/1.73m2 or albuminuria > 30 mg/24 h or albumin-to-creatinine ratio ≥ 30 mg/g). (PDF 226 kb) [file 12882_2018_1021_MOESM3_ESM.pdf]

Supplemental Table 3. Different cutoff values of ferritin and TSAT, adjusted for age and sex, with respect to risk of anemia in CKD patients (based on eGFR<60 ml/min/1.73m^2 or albuminuria >30 mg/24 hours or albumin-to-creatinine ratio ≥ 30 mg/g)

TSAT (%)	HR (95%CI)
<10	**3.07 (1.69-5.57)**
<15	**2.09 (1.43-3.04)**
<20	**1.62 (1.18-2.21)**
<25	**1.60 (1.16-2.23)**
<30	**1.69 (1.14-2.51)**

Ferritin (µg/L)	HR (95%CI)
<20	**2.95 (1.75-4.98)**
<50	**1.83 (1.24-2.69)**
<100	**1.83 (1.34-2.50)**
<200	**1.59 (1.09-2.32)**
<300	1.67 (0.96-2.91)
<500	6.94 (0.97-49.59)

AND TSAT	FERRITIN	<20	<50	<100	<200	<300	<500
<10		2.04 (0.94-4.39)	**2.64 (1.38-5.04)**	**3.07 (1.69-5.57)**	**3.07 (1.69-5.57)**	**3.07 (1.69-5.57)**	**3.07 (1.69-5.57)**
<15		**2.51 (1.34-4.70)**	**2.42 (1.49-3.94)**	**2.53 (1.64-3.91)**	**2.23 (1.52-3.28)**	**2.14 (1.46-3.12)**	**2.13 (1.46-3.10)**
<20		**2.51 (1.40-4.50)**	**2.00 (1.29-3.10)**	**1.92 (1.34-2.78)**	**1.80 (1.30-2.48)**	**1.67 (1.22-2.30)**	**1.61 (1.18-2.21)**
<25		**2.96 (1.73-5.04)**	**2.06 (1.37-3.09)**	**1.83 (1.32-2.52)**	**1.93 (1.40-2.65)**	**1.64 (1.19-2.27)**	**1.63 (1.17-2.25)**
<30		**2.87 (1.68-4.90)**	**1.89 (1.27-2.80)**	**1.86 (1.36-2.56)**	**1.90 (1.36-2.65)**	**1.69 (1.18-2.42)**	**1.76 (1.19-2.60)**

Conditional definitions:

Ferritin <100 µg/L or TSAT <10% with ferritin 100-199 µg/L	**2.33 (1.99-2.73)**
Ferritin <100 µg/L or TSAT <10% with ferritin 100-299 µg/L	**2.33 (1.99-2.73)**
Ferritin <100 µg/L or TSAT <15% with ferritin 100-199 µg/L	**2.39 (2.04-2.80)**
Ferritin <100 µg/L or TSAT <15% with ferritin 100-299 µg/L	**2.37 (2.02-2.78)**
Ferritin <100 µg/L or TSAT <20% with ferritin 100-199 µg/L (FIND-CKD)	**2.35 (1.99-2.77)**
Ferritin <100 µg/L or TSAT <20% with ferritin 100-299 µg/L (FAIR-HF)	**1.92 (1.38-2.66)**
