# Supplementary material for: Association of different iron deficiency cutoffs with adverse outcomes in chronic kidney disease
Source: BMC Nephrol. 2018 Sep 12;19:225. doi: 10.1186/s12882-018-1021-3 (PMC6134584; doi:10.1186/s12882-018-1021-3)
Supplement: Supplementary file 4 — Table S4, Table S5, and Table S6. Showing the association of different cutoff values of ferritin and TSAT, adjusted for age and sex, in CKD patients with eGFR< 60 ml/min/1.73m2 with respect to risk of all-cause mortality, cardiovascular mortality, and anemia, respectively. (PDF 195 kb) [file 12882_2018_1021_MOESM4_ESM.pdf]

Supplemental Table 4. Different cutoff values of ferritin and TSAT, adjusted for age and sex, with risk of all-cause mortality in CKD patients with eGFR<60 ml/min/1.73m^2

**TSAT (%)**	**HR (95%CI)**
<10	**3.23 (1.39-7.51)**
<15	**1.84 (1.03-3.30)**
<20	1.29 (0.80-2.06)
<25	1.39 (0.86-2.23)
<30	**3.39 (1.63-7.05)**

**Ferritin (µg/L)**	**HR (95%CI)**
<20	0.92 (0.23-3.76)
<50	1.48 (0.79-2.76)
<100	1.35 (0.86-2.11)
<200	1.63 (0.95-2.80)
<300	**2.32 (1.06-5.06)**
<500	2.20 (0.54-8.98)

**AND TSAT**	**FERRITIN**	<20	<50	<100	<200	<300	<500
<10		1.21 (0.17-8.81)	2.37 (0.74-7.56)	2.11 (0.77-5.82)	**2.67 (1.07-6.66)**	**2.67 (1.07-6.66)**	**2.67 (1.07-6.66)**
<15		0.74 (0.10-5.31)	1.58 (0.76-3.29)	**1.92 (1.05-3.49)**	**1.92 (1.05-3.47)**	**1.91 (1.05-3.47)**	**1.69 (0.93-3.07)**
<20		0.92 (0.23-3.76)	0.92 (0.40-2.12)	1.16 (0.65-2.08)	**1.64 (1.01-2.64)**	1.49 (0.92-2.40)	1.28 (0.79-2.06)
<25		0.92 (0.23-3.76)	1.16 (0.57-2.34)	1.23 (0.76-1.99)	**1.65 (1.05-2.59)**	**1.73 (1.08-2.75)**	**1.39 (0.87-2.23)**
<30		0.92 (0.23-3.76)	1.48 (0.79-2.76)	1.52 (0.97-2.39)	**2.29 (1.39-3.75)**	**2.93 (1.64-5.23)**	**2.90 (1.49-5.63)**

Conditional definitions:

Ferritin <100 µg/L or TSAT <10% with ferritin 100-199 µg/L	1.42 (0.91-2.22)
Ferritin <100 µg/L or TSAT <10% with ferritin 100-299 µg/L	1.42 (0.91-2.22)
Ferritin <100 µg/L or TSAT <15% with ferritin 100-199 µg/L	1.55 (0.97-2.42)
Ferritin <100 µg/L or TSAT <15% with ferritin 100-299 µg/L	1.55 (0.99-2.41)
Ferritin <100 µg/L or TSAT <20% with ferritin 100-199 µg/L (FIND-CKD)	**1.82 (1.16-2.86)**
Ferritin <100 µg/L or TSAT <20% with ferritin 100-299 µg/L (FAIR-HF)	**1.70 (1.08-2.67)**

Supplemental Table 5. Different cutoff values of ferritin and TSAT, adjusted for age and sex, with risk of cardiovascular mortality in CKD patients with eGFR<60 ml/min/1.73m^2.

TSAT (%)	HR (95%CI)
<10	**5.35 (1.85-15.48)**
<15	2.21 (0.96-5.11)
<20	1.83 (0.92-3.66)
<25	1.80 (0.84-3.87)
<30	**6.20 (1.48-25.94)**

Ferritin (µg/L)	HR (95%CI)
<20	1.08 (0.15-7.89)
<50	1.53 (0.58-4.00)
<100	**2.06 (1.05-4.05)**
<200	1.77 (0.77-4.09)
<300	2.46 (0.75-8.12)
<500	-

AND TSAT	FERRITIN	<20	<50	<100	<200	<300	<500
<10		3.27 (0.43-24.68)	**6.09 (1.83-20.24)**	**5.37 (1.86-15.58)**	**5.37 (1.86-15.53)**	**5.37 (1.86-15.53)**	**5.37 (1.86-15.53)**
<15		1.80 (0.25-13.15)	2.61 (0.79-8.61)	2.45 (0.94-6.34)	**2.55 (1.10-5.91)**	**2.54 (1.09-5.89)**	2.21 (0.96-5.12)
<20		1.08 (0.15-7.89)	1.55 (0.55-4.42)	1.78 (0.80-3.99)	**2.49 (1.24-4.98)**	**2.28 (1.14-4.57)**	1.93 (0.97-3.86)
<25		1.08 (0.15-7.89)	1.30 (0.45-3.73)	1.71 (0.85-3.43)	1.97 (0.98-3.95)	**2.21 (1.05-4.64)**	1.92 (0.90-4.12)
<30		1.08 (0.15-7.89)	1.53 (0.58-4.00)	**2.30 (1.17-4.52)**	**2.96 (1.33-6.56)**	**3.79 (1.46-9.82)**	6.79 (1.62-28.37)

Conditional definitions:

Ferritin <100 µg/L or TSAT <10% with ferritin 100-199 µg/L	**2.06 (1.05-4.05)**
Ferritin <100 µg/L or TSAT <10% with ferritin 100-299 µg/L	**2.06 (1.05-4.05)**
Ferritin <100 µg/L or TSAT <15% with ferritin 100-199 µg/L	**2.32 (1.17-4.61)**
Ferritin <100 µg/L or TSAT <15% with ferritin 100-299 µg/L	**2.32 (1.17-4.60)**
Ferritin <100 µg/L or TSAT <20% with ferritin 100-199 µg/L (FIND-CKD)	**3.27 (1.54-6.77)**
Ferritin <100 µg/L or TSAT <20% with ferritin 100-299 µg/L (FAIR-HF)	**3.04 (1.44-6.38)**

Supplemental Table 6. Different cutoff values of ferritin and TSAT, adjusted for age and sex, with risk of anemia in CKD patients with eGFR<60 ml/min/1.73m^2.

TSAT (%)	HR (95%CI)
<10	**4.80 (1.87-12.31)**
<15	**2.43 (1.29-4.58)**
<20	1.38 (0.82-2.32)
<25	1.17 (0.68-2.00)
<30	1.40 (0.75-2.59)

Ferritin (µg/L)	HR (95%CI)
<20	1.97 (0.61-6.36)
<50	1.32 (0.64-2.72)
<100	1.57 (0.95-2.60)
<200	1.52 (0.82-2.83)
<300	2.39 (0.86-6.62)
<500	2.76 (0.38-19.89)

**AND TSAT**	**FERRITIN**	<20	<50	<100	<200	<300	<500
<10		3.07 (0.71-13.37)	3.14 (0.96-10.29)	**4.80 (1.87-12.31)**	**4.80 (1.87-12.31)**	**4.80 (1.87-12.31)**	**4.80 (1.87-12.31)**
<15		**3.81 (1.15-12.59)**	**3.80 (1.62-8.94)**	**5.25 (2.68-10.29)**	**2.96 (1.59-5.50)**	**2.74 (1.46-5.16)**	**2.43 (1.29-4.58)**
<20		1.97 (0.61-6.36)	**1.52 (0.69-3.35)**	**2.05 (1.16-3.59)**	**1.61 (0.95-2.74)**	**1.42 (0.84-2.40)**	**1.32 (0.78-2.23)**
<25		1.97 (0.61-6.36)	1.26 (0.59-2.67)	1.46 (0.86-2.48)	1.47 (0.88-2.47)	1.20 (0.71-2.02)	1.15 (0.68-1.96)
<30		1.97 (0.61-6.36)	1.32 (0.64-2.72)	1.49 (0.89-2.51)	1.50 (0.88-2.55)	1.44 (0.82-2.53)	1.39 (0.76-2.53)

Conditional definitions:

Ferritin <100 µg/L or TSAT <10% with ferritin 100-199 µg/L	1.58 (0.95-2.60)
Ferritin <100 µg/L or TSAT <10% with ferritin 100-299 µg/L	1.58 (0.95-2.60)
Ferritin <100 µg/L or TSAT <15% with ferritin 100-199 µg/L	1.49 (0.90-2.54)
Ferritin <100 µg/L or TSAT <15% with ferritin 100-299 µg/L	1.44 (0.88-2.37)
Ferritin <100 µg/L or TSAT <20% with ferritin 100-199 µg/L (FIND-CKD)	1.44 (0.87-2.38)
Ferritin <100 µg/L or TSAT <20% with ferritin 100-299 µg/L (FAIR-HF)	1.30 (0.79-2.15)
