# Supplementary material for: Association of different iron deficiency cutoffs with adverse outcomes in chronic kidney disease
Source: BMC Nephrol. 2018 Sep 12;19:225. doi: 10.1186/s12882-018-1021-3 (PMC6134584; doi:10.1186/s12882-018-1021-3)
Supplement: Supplementary file 5 — Table S7, Table S8, and Table S9. Showing the association of different cutoff values of ferritin and TSAT, adjusted for age, sex, hs-CRP, and albumin, in 717 CKD patients (based on eGFR< 60 ml/min/1.73m2 or albuminuria > 30 mg/24 h or albumin-to-creatinine ratio ≥ 30 mg/g) with respect to risk of all-cause mortality, cardiovascular mortality, and anemia, respectively. (PDF 255 kb) [file 12882_2018_1021_MOESM5_ESM.pdf]

Supplemental Table 7. Different cutoff values of ferritin and TSAT, adjusted for age, sex, hs-CRP, and albumin, with risk of all-cause mortality in 717 CKD patients (based on eGFR<60 ml/min/1.73m^2 or albuminuria >30 mg/24 hours or albumin-to-creatinine ratio ≥ 30 mg/g)

**TSAT (%)**	**HR (95%CI)**
<10	**2.87 (1.45-5.68)**
<15	1.40 (0.87-2.26)
<20	1.24 (0.85-1.81)
<25	1.37 (0.95-1.96)
<30	**2.17 (1.33-3.53)**

**Ferritin (µg/L)**	**HR (95%CI)**
<20	0.61 (0.15-2.48)
<50	1.47 (0.91-2.38)
<100	1.08 (0.75-1.55)
<200	1.36 (0.91-2.02)
<300	1.64 (0.92-2.92)
<500	2.87 (0.71-11.62)

**AND TSAT**	**FERRITIN**	<20	<50	<100	<200	<300	<500
<10		1.16 (0.28-4.77)	**2.38 (1.04-5.44)**	**2.54 (1.24-5.20)**	**2.87 (1.45-5.68)**	**2.87 (1.45-5.68)**	**2.87 (1.45-5.68)**
<15		0.72 (0.18-2.90)	1.81 (0.94-3.47)	1.64 (0.95-2.84)	1.57 (0.96-2.55)	1.49 (0.91-2.43)	1.43 (0.88-2.30)
<20		0.65 (0.16-2.62)	1.34 (0.72-2.49)	1.01 (0.63-1.64)	1.44 (0.98-2.01)	1.34 (0.92-1.95)	1.29 (0.89-1.88)
<25		0.61 (0.15-2.49)	1.52 (0.89-2.59)	1.11 (0.75-1.64)	1.41 (0.99-1.99)	**1.52 (1.07-2.17)**	**1.44 (1.00-2.06)**
<30		0.61 (0.15-2.49)	1.56 (0.96-2.52)	1.26 (0.88-1.81)	**1.69 (1.18-2.43)**	**2.04 (1.35-3.08)**	**2.11 (1.32-3.38)**

Conditional definitions:

Ferritin <100 µg/L or TSAT <10% with ferritin 100-199 µg/L	1.12 (0.78-1.60)
Ferritin <100 µg/L or TSAT <10% with ferritin 100-299 µg/L	1.12 (0.78-1.60)
Ferritin <100 µg/L or TSAT <15% with ferritin 100-199 µg/L	1.11 (0.78-1.58)
Ferritin <100 µg/L or TSAT <15% with ferritin 100-299 µg/L	1.09 (0.77-1.55)
Ferritin <100 µg/L or TSAT <20% with ferritin 100-199 µg/L (FIND-CKD)	**1.41 (1.00-2.00)**
Ferritin <100 µg/L or TSAT <20% with ferritin 100-299 µg/L (FAIR-HF)	1.32 (0.93-1.87)

Supplemental Table 8. Different cutoff values of ferritin and TSAT, adjusted for age, sex, hs-CRP, and albumin, with risk of cardiovascular mortality in CKD patients (based on eGFR<60 ml/min/1.73m^2 or albuminuria >30 mg/24 hours or albumin-to-creatinine ratio ≥ 30 mg/g)

**TSAT (%)**	**HR (95%CI)**
<10	**5.50 (2.32-12.99)**
<15	**2.41 (1.23-4.74)**
<20	**1.80 (1.00-3.23)**
<25	1.31 (0.74-2.33)
<30	**2.38 (1.07-5.31)**

**Ferritin (µg/L)**	**HR (95%CI)**
<20	0.88 (0.12-6.41)
<50	1.51 (0.70-3.25)
<100	1.21 (0.69-2.14)
<200	**2.11 (1.01-4.38)**
<300	**4.16 (1.00-17.21)**
<500	-

**AND TSAT**	**FERRITIN**	<20	<50	<100	<200	<300	<500
<10		1.90 (0.26-14.05)	**4.81 (1.70-13.59)**	**5.51 (2.33-13.01)**	**5.50 (2.33-12.99)**	**5.50 (2.33-12.99)**	**5.50 (2.33-12.99)**
<15		1.09 (0.15-7.94)	**3.20 (1.34-7.63)**	**3.02 (1.45-6.28)**	**2.57 (1.28-5.15)**	**2.46 (1.23-4.94)**	**2.46 (1.25-4.83)**
<20		0.93 (0.13-6.78)	2.04 (0.86-4.84)	1.25 (0.60-2.58)	**2.19 (1.22-3.91)**	**1.92 (1.07-3.43)**	**1.88 (1.05-3.35)**
<25		0.88 (0.12-6.44)	1.74 (0.77-3.94)	1.08 (0.58-2.02)	1.41 (0.81-2.47)	1.55 (0.88-2.73)	1.38 (0.77-2.44)
<30		0.88 (0.12-6.44)	1.60 (0.74-3.45)	**1.47 (0.83-2.59)**	**2.38 (1.28-4.43)**	**2.69 (1.30-5.54)**	**2.61 (1.17-5.80)**

Conditional definitions:

Ferritin <100 µg/L or TSAT <10% with ferritin 100-199 µg/L	1.21 (0.69-2.14)
Ferritin <100 µg/L or TSAT <10% with ferritin 100-299 µg/L	1.21 (0.69-2.14)
Ferritin <100 µg/L or TSAT <15% with ferritin 100-199 µg/L	1.22 (0.70-2.14)
Ferritin <100 µg/L or TSAT <15% with ferritin 100-299 µg/L	1.20 (0.69-2.10)
Ferritin <100 µg/L or TSAT <20% with ferritin 100-199 µg/L (FIND-CKD)	**2.04 (1.15-3.63)**
Ferritin <100 µg/L or TSAT <20% with ferritin 100-299 µg/L (FAIR-HF)	**1.87 (1.05-3.33)**

Supplemental Table 9. Different cutoff values of ferritin and TSAT, adjusted for age, sex, hs-CRP, and albumin, with risk of anemia in CKD patients (based on eGFR<60 ml/min/1.73m^2 or albuminuria >30 mg/24 hours or albumin-to-creatinine ratio ≥ 30 mg/g)

TSAT (%)	HR (95%CI)
<10	**2.82 (1.29-6.19)**
<15	**2.31 (1.44-3.69)**
<20	**1.57 (1.06-2.33)**
<25	**1.75 (1.17-2.62)**
<30	**1.78 (1.10-2.87)**

Ferritin (µg/L)	HR (95%CI)
<20	**2.81 (1.37-5.76)**
<50	**1.91 (1.19-3.06)**
<100	**1.52 (1.03-2.25)**
<200	1.24 (0.79-1.94)
<300	1.17 (0.63-2.15)
<500	4.32 (0.60-30.79)

AND TSAT	FERRITIN	<20	<50	<100	<200	<300	<500
<10		2.02 (0.72-5.64)	2.17 (0.87-5.42)	**2.82 (1.29-6.19)**	**2.82 (1.29-6.19)**	**2.82 (1.29-6.19)**	**2.82 (1.29-6.19)**
<15		**2.62 (1.12-6.11)**	**2.59 (1.37-4.91)**	**2.48 (1.42-4.32)**	**2.45 (1.51-3.99)**	**2.36 (1.46-3.82)**	**2.38 (1.49-3.82)**
<20		2.06 (0.89-4.80)	**1.82 (1.02-3.23)**	**1.50 (0.95-2.38)**	**1.61 (1.08-2.42)**	**1.52 (1.02-2.23)**	**1.54 (1.04-2.23)**
<25		**2.59 (1.22-5.48)**	**2.02 (1.21-3.38)**	**1.51 (1.02-2.26)**	**1.81 (1.23-2.65)**	**1.60 (1.08-2.35)**	**1.73 (1.16-2.58)**
<30		**2.59 (1.22-5.48)**	**1.93 (1.19-3.14)**	**1.55 (1.05-2.29)**	**1.72 (1.15-2.57)**	**1.53 (1.00-2.35)**	**1.80 (1.22-2.88)**

Conditional definitions:

Ferritin <100 µg/L or TSAT <10% with ferritin 100-199 µg/L	**1.52 (1.03-2.25)**
Ferritin <100 µg/L or TSAT <10% with ferritin 100-299 µg/L	**1.52 (1.03-2.25)**
Ferritin <100 µg/L or TSAT <15% with ferritin 100-199 µg/L	**1.68 (1.14-2.47)**
Ferritin <100 µg/L or TSAT <15% with ferritin 100-299 µg/L	**1.68 (1.14-2.47)**
Ferritin <100 µg/L or TSAT <20% with ferritin 100-199 µg/L (FIND-CKD)	**1.74 (1.18-2.58)**
Ferritin <100 µg/L or TSAT <20% with ferritin 100-299 µg/L (FAIR-HF)	**1.71 (1.15-2.55)**
